# The Story of the Insane from Year to Year

**Published:** 1906-09-15

**Authors:** 


### THE STORY OF THE INSANE FROM YEAR TO YEAR.

Eighteenth Annual Series.

### HEREFORD COUNTY ASYLUM.

On the first of January there were in residence 527 patients, and at the end of the year the number had risen to 545. There were 79 first admissions; 15 readmissions; 24 discharged recovered; 16 discharged relieved, and 34 died. The total number under treatment was 621, and the average daily number resident was 536. The recoveries were 28.9 per cent. of the admissions, and the deaths were 6.3 per cent. of the average number resident. Of the year's deaths 11 were caused by cerebral and spinal diseases; 6 by tubercular disease; 2 by pneumonia, and 1 by enteric fever. Of the year's admissions various moral causes account for the insanity in 14 instances; and among the physical causes, 42 were hereditary or congenital; 14 were owing to intemperance in drink, and 15 were owing to old age. The Committee and the Medical Superintendent call attention to the number of senile admissions, and to the unnecessary strain on the accommodation which the ever-increasing number of these cases entails. The Committee state that "it never could have been anticipated by the Lunacy Act that simple dotage, and the bodily infirmities of old age should be certified for asylum treatment." No; but it might have been anticipated that the four-shilling capitation grant would have the effect of draining the workhouse of these senile cases and filling the asylums with them; and we feel certain that until that grant is either abolished or diverted the Hereford Committee and the Medical Superintendent will cry in vain. In the meantime the asylum is full and accommodation has to be obtained elsewhere at a much higher rate. Dr. Morrison remarks that the out-door treatment for consumption has been carried out by him for a great many years as well as possible without the aid of special structures, and he now very properly suggests "that some suitable inexpensive out-door shelter" should be provided for tubercular cases. The Commitee bear testimony to the care, attention and skill which have been bestowed on the asylum by the Superintendent, the medical staff, and the officers." The weekly cost per head is 9s.

